# Application of Salicylic Acid Derivative in Modifying the Iron Nutritional Value of Lettuce (*Lactuca sativa* L.)

**DOI:** 10.3390/plants13020180

**Published:** 2024-01-09

**Authors:** Barbara Frąszczak, Renata Matysiak, Marcin Smiglak, Rafal Kukawka, Maciej Spychalski, Tomasz Kleiber

**Affiliations:** 1 Department of Vegetable Crops, Faculty of Agronomy, Horticulture and Bioengineering, Poznan University of Life Sciences, Dąbrowskiego 159, 60-594 Poznan, Poland; barbara.fraszczak@up.poznan.pl; 2 Department of Plant Physiology, Faculty of Agronomy, Horticulture and Bioengineering, Poznan University of Life Sciences, Wołyńska 35, 60-637 Poznan, Poland; renata.matysiak@up.poznan.pl; 3 Poznan Science and Technology Park, Adam Mickiewicz University Foundation, Rubież 46, 61-612 Poznan, Poland or marcin.smiglak@innosil.pl (M.S.); rafal.kukawka@ppnt.poznan.pl (R.K.); maciej.spychalski@ppnt.poznan.pl (M.S.); 4 Innosil Sp. z o.o., Rubież 46, 61-612 Poznan, Poland

**Keywords:** Fe chelate, biofortification, foliar spraying, exogenous salicylic acid

## Abstract

The present experiment addressed the effects of foliar sprays of different iron (Fe) concentrations (mg L^−1^), i.e., 2.8 (Fe I), 4.2 (Fe II), and 5.6 (Fe III), as well as an ionic derivative of salicylic acid (iSal) in two doses (10 and 20 mg L^−1^) on lettuce yield, chlorophyll and carotenoids content, and fluorescence parameters. Chemicals were used individually and in combinations two times, 23 and 30 days after the plants were transplanted. This experiment was carried out in a climate chamber. The Fe and iSal applications generally (except Fe I iSal, 10 mg L^−1^; Fe I iSal, 20 mg L^−1^; and Fe III iSal, 20 mg L^−1^) did not influence the fresh and dry matter content. The concentration of chlorophylls and carotenoids was reduced for all treatments in comparison to the control (without spraying). The Fe content in leaves was promoted in the Fe-treated plants (+70% for Fe III + iSal, 10 mg L^−1^, and Fe I). The iSal treatment promoted the Mn content. For most combinations, the Zn and Cu accumulations, as well as the fluorescence parameters, decreased after the foliar spray applications. Overall, our study revealed the effectiveness of Fe-DTPA chelate, but not iSal, in increasing the Fe content of lettuce grown in soilless cultivation systems.

## 1. Introduction

Lettuce is one of the most widely consumed vegetables worldwide, but its nutritional value has been underestimated. Lettuce is low in calories, fat, and sodium. It is a good source of fibre, iron, folate, and vitamin C. Lettuce is also a good source of various other health-beneficial bioactive compounds, like phenolic compounds and carotenoids [1].

Iron (Fe) is a very important micronutrient for both plants and humans. It participates in processes such as photosynthesis, respiration, and oxygenation [2]. However, about two billion people suffer from anaemia, primarily due to a diet low in Fe [3]. In addition, its phytoavailable concentration (10^−17^ M) does not reach the optimal range for plant growth (10^−9^–10^−4^ M) [4]. It should be further noted that Fe is poorly absorbed by the human body, with only about 14–18% of Fe available in food being bioavailable. [5]. Therefore, one way to solve the above problems may be to increase Fe in plant foods [6]. This condition can be achieved by biofortifying plants with a specific element [7]. A safe way to carry this out is through soilless cultivation, where it is possible to control water availability, pH, and nutrient concentration in the root zone [8]. Biofortification is carried out by increasing the level of a specific element in the nutrient solution. Additionally, in the case of Fe, its bioavailability can be increased by managing the pH in the nutrient solution [9]. Since, as mentioned above, Fe is not easily assimilated by plants and excess Fe can be harmful to plants (DNA and protein damage) and cause stress conditions for plant growth [10], using biostimulants or growth inducers that will alleviate the effects of stress and increase Fe assimilation is most appropriate.

According to the European Council Regulation (EC) No. 2019/1009, certain substances, mixtures, and products of microorganisms have been defined as plant biostimulants, and they can be used as fertilizer products according to the European Union (EU). Their task is to stimulate the nutritional processes of plants independently of the nutrient content of the product for the sole purpose of improving one or more of the following plant or plant rhizosphere characteristics: nutrient use efficiency, tolerance to abiotic factors and stress, quality characteristics, or the availability of limited nutrients in the soil or rhizosphere [11].

Several studies have shown that biostimulants of various origins can improve the uptake of nutrients, including minerals, in plants [12,13]. Among other things, biostimulants improve the plant uptake of Ca, Mg, and K [14]. Plant biostimulants also enhance the ion ratios of Na^+^:Ca^2+^ and Na^+^:Mg^2+^ in lettuce leaves [15].

One natural biostimulant is willow bark. The extract of this herbal raw material contains salicylic acid (SA). One of its characteristics is its strong fungicidal properties [16]. Another way in which salicylic acid and its ionic derivatives, such as choline salicylate, interact with plants is by influencing signalling pathways, leading to the induction of systemic acquired resistance (SAR) [17,18]. In our previous studies, we demonstrated that the derivatization of another well-known plant resistance inducer from the benzothiadiazole group leads to an increased expression of genes encoding pathogenesis-related (PR) proteins [19]. The effectiveness of SAR inductors has been confirmed in practice in various tested crops, where the activation of the immune system was observed by a reduction in the occurrence of diseases [20,21]. Moreover, it was found that the induction of resistance is correlated with the stimulation of plant metabolism, providing lasting beneficial effects to a diverse range of crop plants [22,23].

Some researchers suggest that SA plays an important role not only in protecting plants from disease but also in thermogenesis, abiotic stress and salinity tolerance, DNA damage/repair, seed germination, fruit yield, etc. [24]. According to recent studies, it can also be used for rooting woody (lavender) and semi-woody (chrysanthemum) cuttings [25] due to the presence of indole 3-butyric acid (IBA), as well as maize seedling production under salt stress conditions [26]. There was also determined to be a positive effect of salicylic acid contained in willow extract on some macronutrient uptake in lettuce [15].

The chemical composition of willow extract and the amount of salicylate compounds can vary depending on the age of the plant, the date of harvest of the herbaceous material, tissue, genotype, and species, as well as various environmental factors [27], so it was reasonable to create a synthetic SA with established and stable properties.

The conducted study aimed to evaluate the possibility of iSal (an ionic derivative of salicylic acid) application to modify the iron nutritional status of lettuce (*Lactuca sativa* L. cv. ‘Zeralda’).

## 2. Results and Discussion

### 2.1. The Yield

The spray applications of both Fe and iSal did not affect the fresh weight of lettuce (Table 1). The application of the highest doses of Fe and Fe I iSal (10 mg L^−1^), Fe I iSal (20 mg L^−1^), and Fe III iSal (20 mg L^−1^) increased dry matter yield relative to the control. The other combinations resulted in similar dry matter yields and DM percentages.

In earlier studies, increasing Fe dosage (ranging from 0.9 to 25 mg L^−1^) caused a decrease in the fresh weight of biofortified plants [28]. This was related to Fe toxicity above a certain level of doses used in biofortification [9]. Excess Fe levels in plants cause an increase in reactive oxygen species (ROS) production, oxidative stress responses, and physiological disorders [29]. In the current study, no symptoms of toxicity were observed, which means that the doses used in this experiment were safe for plants [30]. According to Filho et al. [28], for *Cichorium intybus* cultivated in an NFT, the optimal Fe range was from 2.7 to 8.3 mg L^−1^. In the current experiment, the Fe doses applied resulted in a higher dry matter yield and % dry matter content. In particular, the highest Fe dose (Fe III, 5.6 mg L^−1^) resulted in a greater dry mass. Interestingly, in another experiment, the dry matter content (%) in lettuce increased as the Fe dose increased, and in turn, the dry matter yield decreased [31]. It is worth noting that in this study, there was no effect of iSal on the lettuce biomass.

### 2.2. Microelement Content and Uptake

The applied sprays with iron as well as iSal modified the content and uptake of microelements by the lettuce leaves (Table 2). Each of the foliar spray treatments increased the Fe content in the lettuce leaves. The highest amount was obtained for the highest level of Fe with 10 mg L^−1^ iSal (70% more than in the control). However, it is worth noting that spraying only iSal or spraying iSal at a dose of 20 mg L^−1^ with added Fe had less effect on the content of this microelement in the leaves compared to the other foliar spray treatments. Similarly, spraying only Fe significantly increased the Fe uptake by the lettuce plants compared to the control combination, but the application of only iSal did not affect Fe uptake as strongly. Iron bioavailability may be affected by polyphenols due to the high affinity of these compounds for this mineral [32]. This may have been the reason for the reduction in the Fe content in the leaves when a higher dose of iSal was applied. The use of Fe biofortification also proved to be effective in increasing the Fe content in the cultivation of lettuce in some previous studies [7,31,33].

The well-known antagonistic effect of Fe on Mn absorption was not found in the conducted studies. In general, the treatments resulted in a higher Mn content in the leaves and a higher uptake by the leaves compared to the control combination. However, the Mn content of the lettuce leaves and the uptake by the leaves varied widely. The lowest content was obtained for the Fe I iSal 10 mg L^−1^ combination. Also, the treatments of Fe I and Fe II with 10 mg L^−1^ of iSal obtained a lower uptake, similar to that of the control combination. The lettuce treated Fe I and with a 20 mg L^−1^ iSal dose was characterised by the highest Mn content and uptake, significantly higher compared to treating plants with Fe I alone. We can conclude that the low doses of applied Fe with high doses of iSal reduced the Mn content and uptake compared to the control. The application of 10 mg L^−1^ of iSal alone also had a positive effect on the Mn content, as did the application of Fe II and Fe III. Previous studies have shown that foliar Fe application had much less or no effect on Mn uptake and content in soybean [34] and chickpea plants [35]. In chickpeas, foliar Fe and Mn additions increased the average Fe concentration and uptake in shoots. The antagonism of the two elements mainly occurs in the soil fertilization of plants through the negative effect of Fe on the translocation of Mn from the root to the shoot [35]. Our research resulted in a low Fe:Mn ratio. The Fe: Mn ratio varied from 1.6 to 1.0 in the leaves, which resulted from high levels of Mn in the leaves. At the molecular level, excess Mn may prevent the uptake and translocation of other essential elements, including Fe [36]. However, there was no negative effect of high Mn levels on the uptake of Fe and Fe content in the leaves. In another study, the application of two levels of Fe (1 and 2 mmol L^−1^ of Fe, in chelate form) in a nutrient solution also significantly increased the Mn as well as Zn content of lettuce leaves [37]. However, in these studies, the Fe:Mn ratio was much higher at 3.5 and 4.0 for the Fe-enriched nutrient solutions and 2.2 for the control, respectively. This may have been because the plants were fertilised with Fe-enriched nutrient solution all the time.

The Fe treatment significantly reduced the Zn content uptake in the leaves. The content of this microelement was positively affected by spraying iSal at a dose of 20 mg L^−1^. In contrast, spraying Fe alone (without Fe I) or Fe with an iSal dose of 20 mg L^−1^ significantly reduced the Zn content in the leaves. The highest uptake was also noted for the iSal 20 mg L^−1^ combination. However, there were no statistically significant differences between this combination and the control and Fe I and Fe I iSal 10 mg L^−1^ combinations. All the applied sprays except Fe I significantly reduced the Cu content in the leaves compared to the control combination. Most of the treatments also had a negative effect on Cu uptake. The best results were obtained for the Fe I and Fe III iSal 20 mg L^−1^ combinations as well as the control.

One of the biggest problems associated with plant biofortification is the antagonism among some nutrients. The enrichment of plants with one ion reduces the uptake of others [38]. Such a plant response may be due, among other things, to the fact that Fe and other elements share the same membrane transporters, resulting in the competition of iron with other cations [39,40]. It is worth noting that in some studies, an increase in the level of Fe in a medium also contributed to an increase in the level of Zn in lettuce leaves [37]. The effect of Fe on a plant’s Zn and Cu uptake and their content in the leaves also depends on the form of fertilizer used for biofortification. For example, iron-ammonium sulphate increased Zn uptake compared to untreated control plants, and chelates increased the Cu content in African marigolds [41].

In the current study, the biostimulant iSal greatly increased the uptake and content of Mn and Zn in the plants, in contrast to the uptake and content of Fe. This also supports the idea that salicylic acid applications work as biostimulants rather than fertilizers and contribute to the induction of different metabolic pathways beyond providing nutrients to the plant [26].

### 2.3. Chlorophyll and Carotenoid Content

There was a tendency for the chlorophyll (Chl) and carotenoid (Car) content to decrease after the sprays were applied (Table 3). This is especially evident with the Fe sprays combined with iSal at a dose of 20 mg L^−1^. For these combinations, the level of carotenoids was about 25% lower than in the control, and the chlorophyll content was about 28–20% lower. Other studies have also shown that high SA concentrations (1–5 mM) reduce Chl contents in various plant species. The lowest concentration (10^−5^ M) of SA generated the highest values for Chl content for a 60 d-stage *Brassica juncea* [42]. However, the values decreased as the concentration of SA increased and reached below that of the control at the maximum concentration (10^−3^ M) [42]. Also, in wheat and moong seedlings, as the concentration of applied salicylic acid (SA) increased, the Chl content significantly decreased [43]. According to these authors, SA induces an increase in the hydrogen peroxide (H_2_O_2_) content in plants. The increase in oxidative stress can cause a decrease in the total Chl content, or a decrease in the total Chl content can induce oxidative stress with an increase in SA concentration. In addition, to protect the photosynthetic apparatus from oxidative stress, carotenoid levels may be increased. However, this was not observed in the current study.

Fe spraying also reduced the Chl and Car content compared to the control combination. In contrast, in other studies, Fe application (1.02 and 2.02 mmol L^−1^) promoted the Chls and Car content in lettuce [37], and the application of Fe NPs (0, 5, 10, 20 mg L^−1^) resulted in an enhancement of both pigments’ content in Red Sails Lettuce [33]. The increase in the carotenoid content was probably linked to the high ROS-scavenging ability of this antioxidant [37]. The current study clearly showed the multidirectional influence of Fe applications on the Chls and Car content in lettuce.

### 2.4. Fluorescence Parameters

The values of the minimum chlorophyll fluorescence (Fo), the maximum chlorophyll fluorescence (Fm), and the variable fluorescence (Fv) varied widely (Table 4). However, for most combinations, these values were lower compared to the control. Fo is the minimum fluorescence level, assuming that all antenna pigment complexes associated with the photosystem are open (dark-adapted) [44]. An increase in Fo indicates any difficulty and degradation of photosystem II (D1 protein and another part of the PS) or any disruption of energy transfer to the reaction centre [45]. This suggests that the lettuce plants were partially subjected to photosynthetic stress under the applied treatments.

Spraying the applied chemicals mostly lowered the efficiency of the PSII quantum field (lower Fv), which may have resulted in a greater dissipation of energy in the form of heat [46]. However, the lettuce yield was not affected.

In the present study, there was no effect of the applied sprays on the parameters Fv/Fo and Fv/Fm. The lack of differences among the combinations confirms the low sensitivity of these parameters to changes in the photochemical properties of PSII [47].

Additional treatments applied during plant growth can be stress factors for plants, causing photoinhibition and/or damage to the photosynthetic apparatus. One such treatment may be the application of biostimulants or intensive biofortification. In the present study, foliar spray treatments were shown to affect the photochemical efficiency parameters of PSII in different ways (Table 5). The analysis of PSII function, assessed by PSII photochemical efficiency parameters, showed that both Fe biofortification and iSal sprays can lead to chloroplast dysfunction in lettuce leaves.

For most combinations, the applied sprays did not result in a deterioration of the PSII viability index, i.e., a reduction in the value of the PI_abs parameter compared to the control combination. Previous studies have shown that differences in the PI_abs values can be attributed to genetic differences, physiological traits, and environmental conditions [48,49].

One of the protective mechanisms of the photosynthetic apparatus, especially PSII, against stress-induced damage is the slowing down of electron transport from reaction centres to plastoquinones [50,51]. In the conducted study, a significant reduction in the rate of electron transport (ETo/RC) was found for all the combinations compared to the control. At the same time, there was no increase in energy dissipation at the expense of heat (DIo/RC).

The applied treatments caused a decrease in the flow of absorbed energy through one active reaction centre (ABS/RC). There was a similar tendency for changes in the energy uptake by one active reaction centre (TR0/RC)—it decreased significantly as the foliar sprays were used. The TR0/RC changes indicate a decrease in the conversion efficiency of the excitation energy.

## 3. Materials and Methods

### 3.1. Plant Material and Growth Conditions

The experiment was conducted on lettuce cultivation (*Lactuca sativa* L. cv. ‘Zeralda’) in a growth chamber. NEONICA LED 240 (Poland) modules were used as the light source. The photosynthetic photon flux density (PPFD) was 140 µmol m^−2^ s^−1^, with the following share of individual colours: R (red): 111.7 (µmol m^−2^ s^−1^), G (green): 9.7 (µmol m^−2^ s^−1^), and B (blue): 18.6 (µmol m^−2^ s^−1^). The plants were exposed to light for 16 h; the temperature was maintained at 18/17 °C (day/night); and the RH was approximately 60–75%.

The experiment was established in a randomized design with 5 replications (a replication was one single plant). Seedlings were prepared 30 days before the vegetation experiment. The seeds were sown individually on multiple plates filled with standard peat substrate, as recommended for seedling preparation. The seedlings (in the 3–4-leaf phase) were put in drainless pots filled with perlite (V 500 cm^3^). During the whole experiment, the plants were watered to a stable weight.

The plants were fertigated with a nutrient solution (NS) of the following chemical composition (mg dm^−3^): N-NH_4_, <15; N-NO_3_, 160; P-PO_4_, 40; K, 250; Ca, 150; Mg, 50; Fe, 0.58; Mn, 0.33; Zn, 0.21; Cu, 0.08; B, 0.2. It had a pH of 5.50 and an EC of 1.9 mS·cm^−1^. The following fertilisers for hydroponic cultivation were used to prepare the nutrient solution: potassium nitrate (13% N-NO_3_, 38.2% K), calcium nitrate (14.7% N-NO_3_, 18.5% Ca), mono potassium phosphate (22.3% P, 28.2% K), potassium sulphate (44.8% K, 17% S), magnesium sulphate (9.9% Mg, 13% S), manganese sulphate (32.3% Mn), copper sulphate (25.6% Cu), borax (11.3% B), and sodium molybdate (39.6% Mo).

### 3.2. Foliar Application of Fe and iSal

The studied factors were Fe (1. factor) and iSal application (2. factor). The source of iron was Librel FeDP7 DTPA chelate (7% Fe; Royal Brinkman, Poznan, Poland).

For the research, an active substance in the form of an ionic derivative of salicylic acid (iSal), developed by Poznan Science and Technology Park (PSTP) and the Innosil research team, was used. Currently, the active substance is the subject of patent application PCT/PL/2023/050110 [52]. To prepare a working solution for spraying, the ionic derivative of salicylic acid was weighed and dissolved in water in an amount to prepare solutions with concentrations of 10 and 20 mg L^−1^. The published results are part of preliminary studies conducted to file a patent application.

A foliar spray treatment (5 mL per 1 plant) was applied two times, 23 and 30 days after the transplantation to a stable place (24–25 and 26–27 BBCH-scale, respectively). The plants were treated with different chemicals: control (without spraying), iSal (10 and 20 mg L^−1^), and Fe (3 levels, in mg L^−1^: 2.8, 4.2, and 5.6, described, respectively, as Fe-I, Fe-II, and Fe-III) and the mixture. Ten days after the second spraying, the experiment was finished.

### 3.3. Biometrical and Chlorophyll Fluorescence Measurements

On the day of harvest (40th day after transplanting to the stable place, 28–29 BBCH-scale), the following parameters were determined: the weight of the lettuce leaves (the whole head, g), the dry matter yield (after drying for 24 h at 105 °C), and the dry matter content (% DM). The chlorophyll *a* fluorescence was measured using a PAR-FluorPen FP 110D fluorometer (Photon Systems Instruments Company (PSI), Drásov, Czech Republic). All the plants in the experiment were measured. Leaf fragments were shaded with a special leaf clip for 30 min. Then, the OJIP test was conducted to measure the following chlorophyll fluorescence parameters: F_0_—the initial fluorescence, F_M_—the maximum fluorescence intensity, F_V_—the maximum variable fluorescence, F_V_/F_M_—the maximum photochemical quantum PSII after dark adaptation, ABS/RC—the light energy absorbed by the PSII antenna photon flux per active reaction centre, TR_0_/RC—the total energy used to reduce QA by the unit reaction centre of PSII per energy captured by a single active RC, ET_0_/RC—the rate of electron transport through a single RC, DI_0_/RC—non-photochemical quenching per reaction centre of PSII; the total dissipation of energy not captured by the RC in the form of heat, fluorescence, and transfer to other systems, PI_Abs_—the performance index (potential) for energy conservation from excitation to the reduction in intersystem electron acceptors [53].

### 3.4. Chloroplast Pigments

On the day of harvest, the leaf samples from all the tested plants were collected and stored at −20 °C until the analyses. The total chlorophyll and carotenoids content was determined according to the method of Hiscox and Israelstam [54]. The leaf samples (100 mg) were cut into pieces, and pigments were extracted at 65 °C using 5 cm^3^ of dimethyl sulfoxide (DMSO). The optical density of the extracts was measured at 480, 649, and 663 nm. The content of total chlorophyll and carotenoids was calculated following the modified Arnon equations [55] and expressed in mg/g d.m.

### 3.5. Chemical Analysis

All analyses were conducted on the aerial parts of the plants. The samples were dried for 48 h at 45–50 °C to a stable mass and then ground. Before mineralisation, the plant material was dried for 1 h at 105 °C. To analyse the total content of Fe, Mn, Zn, and Cu, the plant material (2.5 g) was dissolved in a mixture of concentrated nitric (ultrapure) and perchloric acids (analytically pure)in a 3:1 ratio (30 cm^3^) [56] (pp. 25–83). After mineralisation, the following measurements were taken: Fe, Mn, Zn, and Cu. These were measured with flame atomic absorption spectroscopy (FAAS) using the Carl Zeiss Jena 5 apparatus (Carl Zeiss Jena, Thornwood, NY, USA). The accuracy of the methods used for the chemical analyses and the precision of the analytical measurements of nutrient levels were tested by analysing the reference material of branched flour (*Pseudevernia furfuracea*), certified by the IRMM (Institute for Reference Materials and Measurements) in Belgium. The procedure was also verified with the LGC7162 reference material (LGC standards), with an average nutrient recovery of 96% (N, P, K, Ca, Mg, Fe, Mn, Zn).

### 3.6. Statistical Analysis

The study was conducted as a one-factor experiment. The results are the averages of five replications. The differences between the means were estimated using Duncan’s test at a significance level of *α* = 0.05. The data were statistically analysed using Statistica 13.3 software (StatSoft Inc., Tulsa, OK, USA).

## 4. Conclusions

The iron and iSal treatments did not affect the linearly fresh and dry matter yields of lettuce, probably because the concentrations of both compounds were within appropriate ranges and had no toxic effects on the plants. The foliar spray of Fe improved the Fe content of the plants and had no negative effect on the Mn content. However, the higher doses of Fe negatively affected the Zn and Cu content when also in combination with iSal. It should be noted that the application of only iSal at a dose of 20 mg L^−1^ did not reduce the Zn content in the plants compared to the other treatments. The study showed that foliar-applied Fe chelate is effective in the biofortification of lettuce.

However, exogenous iSal applied foliarly did not specifically positively affect the uptake or content of micronutrients in the lettuce, except manganese. In addition, iSal at a dose of 20 mg L^−1^ combined with Fe negatively affected the chlorophyll and carotenoid content.

To sum up, we conclude that the foliar spraying of chelate Fe-DTPA may be an alternative for increasing the concentration of this element in lettuce. However, the need for additional applications of exogenous iSal has not been proven in this experiment. This may have been since the lettuce plants were cultivated under optimal growth conditions without any stress factors.

## Figures and Tables

**Table 1:** The influence of the Fe and iSal applications on the yields of plants.

Treatment	Fresh Yield (g)	Dry Matter Yield (g)	% DM
Control	23.22 a *	1.32 a	5.70 a
Fe I	25.17 a	1.62 cd	6.46 abc
Fe II	24.89 a	1.58 bcd	6.35 abc
Fe III	25.19 a	1.64 cd	6.51 bc
iSal 10 mg L^−1^	23.57 a	1.38 ab	5.86 ab
iSal 20 mg L^−1^	23.94 a	1.50 abcd	6.25 abc
Fe I iSal 10 mg L^−1^	24.36 a	1.55 bcd	6.38 abc
Fe II iSal 10 mg L^−1^	24.08 a	1.44 abc	6.01 abc
Fe III iSal 10 mg L^−1^	23.06 a	1.48 abcd	6.42 abc
Fe I iSal 20 mg L^−1^	24.33 a	1.57 bcd	6.50 abc
Fe II iSal 20 mg L^−1^	24.18 a	1.50 abcd	6.20 abc
Fe III iSal 20 mg L^−1^	25.02 a	1.66 d	6.62 c

* Data followed by the same letter do not differ significantly at α = 0.05 for each parameter.

**Table 2:** The influence of the Fe and iSal applications on the content (mg kg^−1^ D.M.) of metallic microelements in lettuce leaves and the uptake (µg·plant^−1^) of them by leaves.

Treatment	Fe	Mn	Zn	Cu
Content (mg kg^−1^ D.M.)				
Control	142.79 a *	132.18 b	39.42 ef	9.26 bc
Fe I	202.75 bc	149.50 cd	37.45 de	9.80 c
Fe II	191.30 bc	163.70 def	30.80 ab	8.23 a
Fe III	197.30 bc	163.67 def	30.67 ab	8.10 a
iSal 10 mg L^−1^	177.27 bc	165.40 ef	37.37 de	8.73 ab
iSal 20 mg L^−1^	176.17 bc	149.60 cd	41.53 f	7.90 a
Fe I iSal 10 mg L^−1^	192.20 bc	117.70 a	37.23 de	8.23 a
Fe II iSal 10 mg L^−1^	193.00 bc	143.23 bc	37.87 de	8.67 ab
Fe III iSal 10 mg L^−1^	204.70 c	151.60 cde	34.70 cd	8.55 ab
Fe I iSal 20 mg L^−1^	172.70 b	173.15 f	29.05 a	8.30 a
Fe II iSal 20 mg L^−1^	175.80 bc	158.55 de	31.40 ab	8.40 ab
Fe III iSal 20 mg L^−1^	183.95 bc	162.15 def	32.45 bc	8.80 ab
Uptake (µg·plant^−1^)				
Control	210.34 a	194.71 ab	58.06 cde	13.63 bc
Fe I	323.26 d	237.90 cdef	59.69 de	15.63 d
Fe II	301.39 cd	258.36 efg	48.59 ab	13.00 ab
Fe III	322.25 d	268.06 fg	50.11 ab	13.24 abc
iSal 10 mg L^−1^	245.20 ab	228.96 cde	51.73 abc	12.06 a
iSal 20 mg L^−1^	263.16 bc	223.63 bcd	62.00 e	11.82 a
Fe I iSal 10 mg L^−1^	298.56 cd	182.50 a	57.83 cde	12.75 ab
Fe II iSal 10 mg L^−1^	279.06 bcd	206.66 abc	54.75 bcd	12.48 ab
Fe III iSal 10 mg L^−1^	302.64 cd	224.76 bcd	51.38 abc	12.61 ab
Fe I iSal 20 mg L^−1^	272.88 bcd	273.63 g	45.91 a	13.12 abc
Fe II iSal 20 mg L^−1^	276.41 bcd	248.51 defg	49.19 ab	13.17 abc
Fe III iSal 20 mg L^−1^	304.35 cd	268.87 fg	53.63 bcd	14.54 cd

* Data followed by the same letter do not differ significantly at α = 0.05 for each parameter.

**Table 3:** The influence of the Fe and iSal applications on chlorophyll and carotenoids content.

Treatment	Chlorophyll *a* [mg g^−1^ d.m.]	Chlorophyll *b* [mg g^−1^ d.m.]	Chlorophyll *a + b* [mg g^−1^ d.m.]	Carotenoids [mg g^−1^ d.m.]
Control	11.16 e *	3.38 c	14.54 e	14.55 e
Fe I	9.60 bcd	2.93 abc	12.53 bcd	12.54 bcd
Fe II	10.94 de	3.26 c	14.20 de	14.20 de
Fe III	10.44 de	3.30 c	13.74 de	13.74 de
iSal 10 mg L^−1^	10.21 cde	3.00 bc	13.21 cde	13.21 cde
iSal 20 mg L^−1^	10.27 cde	3.05 bc	13.32 cde	13.33 cde
Fe I iSal 10 mg L^−1^	10.38 cde	2.93 abc	13.31 cde	13.31 cde
Fe II iSal 10 mg L^−1^	10.23 cde	3.02 bc	13.26 cde	13.26 cde
Fe III iSal 10 mg L^−1^	9.85 bcde	3.00 bc	12.85 bcde	12.86 bcde
Fe I iSal 20 mg L^−1^	8.72 ab	2.59 ab	11.31 ab	11.31 ab
Fe II iSal 20 mg L^−1^	8.10 a	2.47 a	10.57 a	10.57 a
Fe III iSal 20 mg L^−1^	9.00 abc	2.65 ab	11.65 abc	11.65 abc

* Data followed by the same letter do not differ significantly at α = 0.05 for each parameter.

**Table 4:** The influence of the Fe and iSal sprays on chlorophyll fluorescence parameters.

Treatment	Fo	Fm	Fv	Fv/Fo	Fv/Fm
Control	6 525 ef *	48 587 e	42 062 e	6.45 a	0.87 a
Fe I	6 892 f	44 185 cde	38 925 cde	5.65 a	0.88 a
Fe II	5 240 ab	36 513 a	32 437 a	6.19 a	0.89 a
Fe III	5 541 abc	40 018 abc	34 477 abc	6.23 a	0.86 a
iSal 10 mg L^−1^	6 018 cde	43 344 bcd	37 326 bcd	6.20 a	0.86 a
iSal 20 mg L^−1^	5 517 abc	40 253 abc	34 736 abc	6.30 a	0.86 a
Fe I iSal 10 mg L^−1^	5 294 ab	38 115 a	32 821 a	6.19 a	0.86 a
Fe II iSal 10 mg L^−1^	6 209 e	44 226 cde	38 017 bcde	6.13 a	0.86 a
Fe III iSal 10 mg L^−1^	5 359 ab	39 003 ab	33 644 ab	6.28 a	0.86 a
Fe I iSal 20 mg L^−1^	6 161 de	46 068 de	39 908 de	6.48 a	0.87 a
Fe II iSal 20 mg L^−1^	5 664 bcd	40 718 abc	35 055 abc	6.19 a	0.86 a
Fe III iSal 20 mg L^−1^	5 035 a	36 929 a	31 894 a	6.32 a	0.86 a

* Data followed by the same letter do not differ significantly at α = 0.05 for each parameter.

**Table 5:** The influence of the Fe and iSal sprays on chlorophyll fluorescence parameters.

Treatment	Pi_Abs	ABS/RC	TRo/RC	ETo/RC	DIo/RC
Control	10.12 abc*	1.57 d	1.36 c	0.95 c	0.21 c
Fe I	11.41 c	1.31 bc	1.13 ab	0.73 ab	0.17 a
Fe II	10.54 bc	1.20 ab	1.11 ab	0.65 a	0.17 a
Fe III	11.44 c	1.14 a	1.05 ab	0.69 ab	0.16 a
iSal 10 mg L^−1^	9.04 ab	1.33 bc	1.15 ab	0.75 ab	0.18 ab
iSal 20 mg L^−1^	9.41 abc	1.26 abc	1.09 ab	0.71 ab	0.17 a
Fe I iSal 10 mg L^−1^	9.73 abc	1.17 ab	1.01 a	0.65 a	0.16 a
Fe II iSal 10 mg L^−1^	8.23 a	1.39 c	1.19 b	0.77 b	0.20 bc
Fe III iSal 10 mg L^−1^	10.44 bc	1.20 ab	1.03 ab	0.69 ab	0.16 a
Fe I iSal 20 mg L^−1^	9.40 abc	1.33 bc	1.15 ab	0.76 b	0.18 ab
Fe II iSal 20 mg L^−1^	9.48 abc	1.27 abc	1.09 ab	0.71 ab	0.18 ab
Fe III iSal 20 mg L^−1^	9.89 abc	1.21 ab	1.04 ab	0.68 ab	0.17 a

* Data followed by the same letter do not differ significantly at α = 0.05 for each parameter.

## Data Availability

Data are contained within the article.
